# Bacterial contamination and antimicrobial susceptibility patterns of intensive care units medical equipment and inanimate surfaces at Ayder Comprehensive Specialized Hospital, Mekelle, Northern Ethiopia

**DOI:** 10.1186/s13104-019-4658-5

**Published:** 2019-09-23

**Authors:** Addis Darge, Atsebaha Gebrekidan Kahsay, Haftamu Hailekiros, Selam Niguse, Mahmud Abdulkader

**Affiliations:** 1 Medical Laboratory Technology Department, Bahirdar College of Health Sciences, Bahidar, Ethiopia; 2 Medical Microbiology and Immunology Unit, Institute of Biomedical Sciences, College of Health Science, Mekelle University, PO. Box: 1871, Mekelle, Ethiopia

**Keywords:** Bacterial contamination, Medical equipment, Inanimate surfaces, Ethiopia, Antimicrobial susceptibility pattern

## Abstract

**Objective:**

To determine bacterial contaminants and their antimicrobial susceptibility patterns from medical equipment and inanimate surfaces.

**Results:**

Of 130 swabs, 115 (88.5%) swabs were culture positive, of which contaminated medical equipment and inanimate surfaces account 70 (83.3%) and 45 (97.8%), respectively. All the swabs collected from sphygmomanometer, bedside table, computer and computer standing tables were 100% contaminated with bacteria. From the culture-positive swabs, a total of 171 bacterial isolates were identified, out of which 117 (68.4%) and 54 (31.6%) isolates were gram-positive and gram-negative, respectively. Most isolates (82%) were resistant to ampicillin and 13%, 8.6%, and 14% was observed in ciprofloxacin, gentamicin, and tetracycline respectively. Multi-drug resistant was observed in *Escherichia coli* (72.7%) and *Staphylococcus aureus* (58.7%).

## Introduction

Intensive care unit (ICU) acquired infections are global public health concern [1]. Bacterial contamination of the medical equipment and inanimate surfaces used in the ICU put ICU admitted patients (especially those with an underline disease, impaired immunity and with invasive procedures) at higher risk for hospital-acquired infection [2]. Non-critical medical equipment and inanimate surfaces have a capacity to harbor bacteria for a long period of time and can become into contact with patients and medical personnel during disease management [3–6].

The prevalence of ICU acquired infections in developed countries is indicated to be 5–10%, while their prevalence is shown to exceed 2–20 times higher in developing countries [7]. A high incidence (> 50%) of ICU acquired infections are reported as compared with other wards (10%) [1]. This might be due to invasive procedures, presence of debilitating patients, prolonged stays and emerging of multidrug-resistant bacteria [8–10].

Studies have reported that contaminated medical equipment and inanimate surfaces were highly associated with ICU acquired infections [11–16]. Although few studies were carried out in the Hospitals in Ethiopia, most studies were focusing on other wards and those performed in intensive care units were done on single or few medical equipment and inanimate surfaces [17–19]. Moreover, there is no study done in the study setting related to contamination of ICU medical equipment and inanimate surfaces. Therefore, this study aimed at determining bacterial contaminates and antimicrobial susceptibility patterns from medical equipment and inanimate surfaces in the ICUs of Ayder Comprehensive Specialized Hospital, Northern Ethiopia.

## Main text

### Materials and methods

#### Study area, design and period

A hospital-based cross-sectional study was conducted from October 2016 to June 2017 at Ayder Specialized Comprehensive Hospital, Mekelle, Northern Ethiopia. Mekelle city is situated 783 km North of Addis Ababa, the capital of Ethiopia. It is the largest hospital serving for more than 4.1 million people of the region and the neighboring regions. The hospital has adult, neonatal, and pediatric ICU health services.

#### Sampling technique

All non-critical medical equipment and inanimate surfaces that had frequent contact with patients and/or healthcare providers during the study period were included. A total of 130 swabs from medical equipment and inanimate surfaces (55 swabs from Adult, 44 swabs from pediatric and 31 swabs from neonatal) were collected using purposive sampling technique.

#### Sample collection, handling, and transport

Sterile cotton-tipped applicator sticks, moistened with sterile normal saline, was used to collect swab specimen. Swab samples were taken using aseptic techniques with the presence of a spirit lamp. All swab samples were inserted in a separate sterile test tube, labeled and transported to the Medical Microbiology and Immunology Laboratory, Mekelle University, using icebox within 1 h and processed immediately [20].

#### Laboratory processing

##### Bacterial culture and identification

Swabs were inoculated on to Blood agar, MacConkey agar and Mannitol salt agar (Oxoid Ltd, UK). The inoculated plates were incubated at 37 °C for 24 h. The inoculated plates were inspected after 24 h of incubation and bacterial isolates from culture-positive plates were identified at the species level by their colony morphology, gram-staining and biochemical characteristics (catalase, coagulase, urease, indole, oxidase, citrate utilization, glucose and lactose fermentation, gas and hydrogen sulfide production) [20].

##### Antimicrobial susceptibility testing

Antimicrobial susceptibility testing was performed using modified Kirby–Bauer disk diffusion according to the Clinical and Laboratory Standards Institute (CLSI), guideline [21]. Bacterial isolates were tested for commonly used antibiotics including: amoxicillin–clavulanic acid (AMC, 30 mcg), ampicillin (AMP, 10 mcg), ceftriaxone (CTR, 30 mcg), ciprofloxacin (CIP, 5 mcg), chloramphenicol (C, 30 mcg), gentamicin (GEN, 10 mcg) and tetracycline (TE, 30 mcg), cefoxitin (CX, 30 mcg), erythromycin (E, 15 mcg), penicillin G (P, 10 units), amikacin (AK, 30 mcg), cefotaxime (CTX, 30 mcg), nalidixic acid (NA, 30 mcg) (HIMEDIA, Company).

#### Data analysis

Data were entered into Microsoft Excel. Data were imported and analyzed using Statistical Package for Social Sciences (SPSS) software version 21.0. Descriptive statistics were computed and results were summarized by using tables.

#### Quality assurance

Aseptic techniques were used in all the steps of specimen collection and inoculation to minimize contamination. Specimens were collected in the presence of sprit lamp to prevent bacterial contamination from air. Reagents and antimicrobial discs were checked for expiry date. Sterility of culture media was carried out by incubating 5% of the prepared media prior to inoculation. *Escherichia coli* (ATCC 25922), *Staphylococcus aureus* (ATCC 25923) and *Pseudomonas aeruginosa* (ATCC 27853) reference strains were used to control the performance of culture media and antibiotic discs. All results were checked accuracy and cleaned after entering to SPSS.

#### Operational definition

*Non*-*critical medical equipment* is equipment used for the diagnosis and management of the patient in ICU such as sphygmomanometer, stethoscope, and thermometer.

*The inanimate surface* is a surface of the material used in ICU during providing patient care such as bedside tables, mattress, computers, and computer standing tables.

### Results

#### Bacterial contamination of medical equipment and inanimate

A total of 130 swabs (84 from medical equipment and 46 from inanimate surfaces) were collected and inoculated to culture media and all the specimens were analysed. From the total analyzed swabs, bacterial growth was identified in 115 (88.5%) of the swab specimens. Bacterial contaminants were isolated in 70 (83.3%) and 45 (97.8%) from medical equipment and inanimate surfaces, respectively. Bacterial contaminations were statistically significant with the medical and inanimate surfaces bacterial contamination (P-value = 0.022, X^2^ = 14.843). All the swabs collected from sphygmomanometer, bedside table, computer and computer standing tables were 100% contaminated with bacteria. From the culture-positive swabs, a total of 171 bacterial isolates were identified, out of which 117 (68.4%) were gram-positive and 54 (31.6%) were gram-negative bacteria. From the total bacterial isolates in medical equipment and inanimate surfaces, coagulase-negative staphylococcus 53 (34.9%), *Staphylococcus aureus* 40 (26.3%), *Citrobacter freundii* 14 (9.2%) and *Klebsiella pneumoniae* 10 (8%) were the most commonly isolated bacteria. From the contaminated medical equipment samples, 32 (34.8%) CoNS, 23 (25%) *S. aureus* and 13 (14.1%) *C. fraundii*, whereas from the inanimate surface swab samples, 28 (35.4%) CoNS, 23 (29.1%) *S. aureus* and 5 (6.3%) *K. pneumonia* was the most commonly isolated bacteria (Table 1).

**Table 1:** Profile and distribution of isolated bacteria from medical equipment at Ayder Specialized Comprehensive Hospital October 2016 to June 2017

Bacterial isolates	Stethoscope parts			Thermometers		Sphygmomanometer		Mattresses (n = 6)	Bedsides		Computers			CS table (n = 15)	Total
	Ear (n = 25)	Tubing (n = 17)	Diaphragm (n = 19)	TM (n = 6)	TS (n = 6)	SC (n = 10)	SB (n = 8)		BTT (n = 10)	BTB (n = 9)	CM (n = 7)	CE (n = 10)	CS (n = 9)		
Adult ICU															
CoNS	4	3	6	0	0	0	0	1	2	2	2	2	1	1	24
*S. aureus*	7	4	3	0	0	2	1	2	2	1	0	2	1	2	27
*K. pneumoniae*	0	0	1	0	1	2	1	0	0	1	0	0	0	0	6
*E. coli*	1	0	2	1	0	0	1	0	0	0	0	0	0	1	6
Total	12	7	12	1	1	4	3	3	4	4	2	4	2	4	63
Pediatric ICU															
CoNS	1	1	0	0	0	3	1	2	1	2	0	0	2	2	15
*S. aureus*	1	1	0	0	0	0	1	1	2	1	1	2	0	0	10
*C. freundii*	3	3	2	2	2	0	0	0	1	0	0	0	0	1	14
*E. aerogenes*	1	1	1	0	0	3	3	0	0	0	0	0	0	0	9
*S. typhi*	0	0	0	0	0	0	0	0	0	1	1	0	0	0	2
*P. vulgaris*	1	0	0	0	0	0	0	0	0	0	0	0	1	0	2
*E. coli*	0	1	1	0	0	0	0	0	2	1	0	0	0	0	5
Total	7	7	4	2	2	6	5	3	6	5	2	2	3	3	57
Neonatal ICU															
CoNS	3	2	3	3	2	NA	NA	NA	NA	NA	2	2	1	3	21
*S. aureus*	1	1	0	0	1	NA	NA	NA	NA	NA	1	0	2	3	9
*C. fraundii*	1	0	0	0	0	NA	NA	NA	NA	NA	0	0	0	0	1
*K. pneumoniae*	1	0	0	0	0	NA	NA	NA	NA	NA	0	1	1	2	5
Total	6	3	3	3	3	NA	NA	NA	NA	NA	3	3	4	8	36

BTB: bedside table bottom; BTT: bedside table top; CE: computer Enter key; CM: computer mouse; CoNs: Coagulase negative *staphylococcus*; CS table: computer standing table; CS: computer space bar; Diaphragm: stethoscope diaphragm; Ear: stethoscope ear piece; NA: none applicable; TM: thermometer mercury; TS: thermometer side; SB: sphygmomanometer bulb; SC: sphygmomanometer cuff; Tubing: stethoscope tubing

#### Antimicrobial susceptibility patterns of the bacterial isolates

The antimicrobial susceptibility tests of the isolates revealed varying ranges of resistance to the tested antimicrobial agents. More than 50% of *Staphylococcus* species were found to be resistant to penicillin G, erythromycin, amoxicillin–clavulanic acid, ampicillin, cefoxitin. Methicillin-resistant *Staphylococcus aureus* was observed in 34 (73.9%) of the isolates. More than 74% of gram-negative rods were resistant to amoxicillin–clavulanic acid, ampicillin, and nalidixic acid. Moreover, above 63% of *E. coli* isolates were resistant to amoxicillin–clavulanic acid, ceftriaxone, ampicillin, cefotaxime, and nalidixic acid (Table 2). Multidrug resistance was observed in 17 (28.3%) of CoNS, 27 (58.7%) of *S. aureus*, 3 (20%) of *C. freundii* and 8 (72.7%) of *E. coli* (Table 3).

**Table 2:** Antimicrobial susceptibility pattern of gram positives and negative from the medical inanimate surfaces at Ayder Specialized Comprehensive Hospital October 2016 to June 2017

Isolates (N)	Pattern	E	P	AMC	CIP	CTR	TE	C	GEN	AMP	CX	Total (%)
CoNS (60)	S	20 (33.3)	24 (40)	22 (37.2)	46 (44)	34 (57.6)	52 (88.1)	41 (72.8)	50 (83)	4 (6.7)	NA	47.4
	I	10 (16.7)	1 (1.7)	0	5 (8.4)	14 (22)	0	4 (6.7)	4 (6.7)	0	NA	6.7
	R	30 (50)	35 (53.3)	38 (62.7)	9 (13.5)	12 (20.3)	8 (11.8)	15 (23.7)	6 (10.1)	56 (93.2)	NA	42.5
*S. aureus* (46)	S	8 (17.4)	9 (19.5)	17 (36.9)	31 (67.4)	19 (41.3)	36 (78.2)	31 (67.4)	35 (76)	5 (10.8)	12 (26.1)	40.5
	I	15 (32.6)	3 (6.5)	0	4 (8.6)	14 (30.4)	3 (6.5)	3 (6.5)	1 (2.1)	0	0	8.8
	R	23 (50)	34 (73.9)	29 (63)	11 (23.9)	13 (28.2)	7 (15.2)	12 (26)	10 (21.7)	41 (89.1)	34 (73.9)	50.5
Total (106)	S	28 (26.4)	33 (31.1)	39 (36.8)	77 (72.6)	53 (50)	88 (83)	75 (70.8)	85 (80.2)	9 (8.5)	12 (26.1)	44.9
	I	25 (23.6)	4 (3.8)	0	9 (8.5)	28 (26.4)	3 (2.8)	7 (6.6)	5 (4.7)	0	0	7.4
	R	53 (50)	69 (65.1)	67 (63.2)	20 (18.9)	25 (23.6)	15 (14.2)	26 (24.5)	16 (15.1)	97 (91.5)	34 (73.9)	47.9

Isolates	Pattern	AMC	CIP	CTR	TE	C	GEN	AMP	CTX	NA	AK	Total (%)
*C. freundii* (15)	S	0	14 (93.3)	15 (100)	14 (93.3)	15 (100)	15 (100)	2 (13.3)	15 (100)	0	12 (80)	68
	I	2 (13.3)	0	0	1 (6.6)	0	0	1 (6.6)	0	0	0	2.6
	R	13 (86.6)	1 (6.6)	0	0	0	0	12 (80)	0	15 (100)	3 (20)	29.3
*K. pneumoniae* (11)	S	0	9 (81.8)	4 (36.3)	6 (54.5)	5 (33.3)	11 (100)	1 (9)	2 (18.1)	2 (18.1)	8 (72.7)	42.3
	I	1 (9)	1 (9)	1 (9)	2 (18.1)	1 (9)	0	0	0	4 (36.3)	2 (18.1)	10.8
	R	10 (90.9)	1 (9)	6 (54.5)	3 (27.2)	5 (45.4)	0	10 (90.9)	9 (100)	5 (45.4)	1 (9)	47.2
*E. aerogens* (9)	S	0	8 (88.9)	9 (100)	9 (100)	9 (100)	9 (100)	0	9 (100)	0	8 (88.9)	67.8
	I	6 (66.6)	1 (11.1)	0	0	0	0	0	0	0	0	7.8
	R	3 (33.3)	0	0	0	0	0	9 (100)	0	9 (100)	1 (11.1)	24.4
*P. vulgaris* (2)	S	0	2 (100)	1 (50)	1 (50)	2 (100)	2 (100)	0	1 (50)	2 (100)	2 (100)	65
	I	1 (50)	0	0	0	0	0	0	1 (50)	0	0	10
	R	1 (50)	0	1 (50)	1 (50)	0	0	2 (100)	0	0	0	25
*E. coli* (11)	S	0	8 (72.7)	3 (27.3)	6 (50)	8 (72.7)	7 (63.75)	3 (25)	3 (27.3)	3 (27.3)	4 (36.4)	40.2
	I	2 (18.2)	1 (9.1)	0	2 (18.2)	0	3 (27.3)	0	0	1 (9.1)	5 (45.4)	12.3
	R	9 (81.8)	2 (18.2)	8 (72.7)	3 (27.3)	3 (27.3)	1 (9.1)	8 (72.7)	8 (72.7)	7 (63.7)	2 (18.2)	46.4
Total (48)	S	0	42 (84)	33 (66)	39 (78)	41 (82)	45 (90)	7 (14)	30 (60)	7 (14)	35 (70)	55.8
	I	13 (26)	4 (8)	1 (2)	4 (8)	1 (2)	4 (8)	1 (2)	2 (4)	5 (10)	8 (16)	8.6
	R	37 (74)	4 (8)	16 (32)	7 (14)	8 (16)	1 (2)	42 (84)	18 (36)	38 (76)	7 (14)	35.6

AMC: amoxicillin–clavulanic; AMP: ampicillin; CX: cefoxitin; CRT: ceftriaxone; C: chloramphenicol; CIP: ciprofloxacin; CoNS: coagulase negative *staphylococcus*; E: erythromycin; GEN: gentamicin; I: intermediate; P: penicillin G; R: resistant; S: sensitive; TE: tetracycline

**Table 3:** Multidrug resistant (MDR) pattern of isolated bacteria from medical equipments and inanimate surfaces at Ayder Specialized Comprehensive Hospital October 2016 to June 2017

Bacterial species	Resisted antimicrobial agents		No. bacteria (%) for MDR	Bacterial species	Resisted antimicrobial agents		No. bacteria (%) for MDR
	No.	Type			No.	Type	
Coagulase negative *staphylococcus*	R4	E, AMC, CIP, AMP	1 (1.7)	*S. aureus*	R3	CIP, GEN, AMP	1 (2.2)
	R5	E, P, AMC, C, AMP	3 (5)			E, AMP, CX	1 (2.2)
		p, AMC, C, GEN, AMP	2 (3.3)		R4	E, P, AMP, CX	4 (8.7)
		p, AMC, CIP, TE, AMP	2 (3.3)			AMC, C, AMP, CX	1 (2.2)
		E, AMC, CIP, TE, AMP	1 (1.7)		R5	P, AMC, C, AMP, CX	1 (2.2)
		E, P, CIP, C, AMP	1 (1.7)			E, P, AMC, AMP, CX	3 (6.5)
	R6	E, P, AMC, C, CTR, AMP	2 (3.3)			P, AMC, CTR, AMP, CX	1 (2.2)
		E, P, AMC, CTR, GEN, AMP	1 (1.7)		R6	P, CIP, CTR, C, AMP, CX	1 (2.2)
		E, P, AMC, CTR, C, AMP	1 (1.7)			E, P, AMC, CTR, AMP, CX	1 (2.2)
	R7	E, P, AMC, CTR, TE, C, AMP	1 (1.7)			E, P, AMC, C, AMP, CX	2 (4.3)
		E, P, AMC, CIP, CTR, GEN, AMP	1 (1.7)			E, AMC, C, GEN, AMP, CX	1 (2.2)
	R9	E, P, AMC, CIP, CTR, TE, C, GEN, AMP	2 (3.3)		R7	E, P, AMC, CTR, C, AMP, CX	2 (4.3)
	Total		17 (28.3)			p, AMC, CIP, CTR, GEN, AMP, CX	3 (6.5)
*E. coli*	R3	CTR, CTX, AK	1 (9.1)			E, P, CIP, CTR, C, AMP, CX	1 (2.2)
	R5	AMC, CTR, AMP, CTX, NA	2 (18.2)			E, P, AMC, TE, C, AMP, CX	1 (2.2)
		AMC, CTR, AMP, CTX, AK	1 (9.1)		R8	E, P, AMC, CIP, TE, GEN, AMP, CX	1 (2.2)
		AMC, CTR, C, AMP, CTX	1 (9.1)		R9	E, P, AMC, CIP, CTR, TE, GEN, AMP, CX	2 (4.3)
	R6	AMC , CIP, CTR, C, CTX, NA	1 (9.1)		R10	E, P, AMC, CIP, CTR, TE, C, GEN, AMP, CX	1 (2.2)
	R7	AMC, CTR, TE, C, AMP, CTX, NA	1 (9.1)		Total		27 (58.7)
	R9	AMC, CIP, CTR, TE, C, GEN, MP, CTX, NA	1 (9.1)	*C. freundii*	R4	AMC, AMP, NA, AK	3 (20)
	Total		8 (72.7)		Total		3 (20)

P: penicillin; CX: cefoxitin; E: erythromycin; AMC: amoxicillin–clavulanic acid; AMP: ampicillin; CTR: ceftriaxone; CIP: ciprofloxacin; C: chloramphenicol; GEN: gentamicin; TE: tetracycline; AK: amikacin; CTX: cefotaxime; NA: nalidixic acid, R3; R4; R5; R6; R7; R8; R9; R10; R11; resistant to (3, 4, 5, 6, 7, 8, 9, 10, 11 antimicrobial agents), respectively, MDR; non-susceptible to ≥ 1 agent in ≥ 3 antimicrobial categories

### Discussions

In this study, the overall bacterial contamination of noncritical medical equipment and inanimate surfaces were high. In addition to this, in this study, similar bacteria were isolated from swab samples taken from parts of some non-critical medical equipment and inanimate surfaces indicating cross-contamination. The predominant isolate was CoNS. With regard to the antimicrobial susceptibility pattern, the isolates showed a significant resistance pattern.

The findings of this study were compared with other similar studies. This finding is in line with the studies conducted from Ethiopia [17] and Asia [15, 22]. On the other hand, our finding is higher than another study from Ethiopia [19], sub-Saharan Africa [5, 16, 22, 23], Asia [13, 14, 24–26], Brazil [27], Italy [28] and UK [12]. On the contrary, this finding is lower than the other study from Brazil [29]. The difference might be attributed to the frequency of medical equipment and inanimate surfaces decontamination, types of decontaminants used, improper health professional practice, nature of the medical equipment and inanimate surfaces to harbor bacteria [12–14, 22, 24–28]. This might be through bacterial cross-contamination of surfaces. This was similarly observed in a study conducted in the UK [12].

The predominant bacteria isolated were CoNS. Bacterial contamination of stethoscope in this study was in-line with the study results reported from sub-Saharan Africa [5, 16, 23] and Asia [13, 24]. On the other hand, this finding is lower than the studies from Ethiopia [17], Asia [15, 22] and Brazil [29], but higher than studies from Ethiopia [19], Asia [14, 25, 26], Brazil [27, 29], Italy [28] and UK [12]. The bacterial contamination in a sphygmomanometer, was in-line with a study done in the UK [12] but higher than the studies from Nigeria [16], New York [30], Australia [31], France [32] and Iraq [33]. Considering inanimate surfaces, the contamination profile of bedside table was in-line with studies conducted in Brazil [29]. However, our findings from the computer table were higher than the studies done at sub-Saharan Africa [34] and Iraq [35]. Bacterial contamination of computer in this study was in-line with the studies conducted in Ethiopia [36], Egypt [37], and Pakistan [38] and higher than from sub-Saharan Africa [39] and Germany [40]. This might be attributed to the number of user’s and prolong the survival time of bacteria on plastic surfaces [39, 40], and the proximity of the equipment or surface to the patient [29, 35], the differences in sample size and method [16, 17].

With regard to the antimicrobial susceptibility pattern of the isolated bacteria to commonly prescribed antibiotics in our study area, the resistance of CoNS to erythromycin and penicillin G was higher than the studies conducted from Ethiopia [17, 19, 36], but lower than a study from sub-Saharan Africa [34]. In addition to this, in this study, *S. aureus* has shown resistant to ciprofloxacin and gentamycin, which is higher than the studies conducted from Ethiopia [17, 36], but, lower from sub-Saharan Africa [23, 34] and Asia [22, 26]. The characteristic multidrug resistance observed in this study is in line a study from Ethiopia [36], sub-Saharan Africa [34] and Asia [22]. On the other hand, lower multidrug resistance was observed in other studies from Ethiopia [12, 17], sub-Saharan Africa [23] and Asia [26]. The inconsistent drug-resistant and multidrug-resistant patterns observed might be due to variations in geographic areas, hospital environmental conditions, inappropriate administration of antimicrobial drugs, self-medication practice [17, 22, 23, 26, 32, 34, 36].

### Conclusion

In this study, high bacterial contamination was observed in medical equipment and inanimate surfaces. CoNS, *S. aureus*, *C. freundii*, *K. pneumonia*, and *E. coli* were the most commonly isolated bacteria. Resistance to amoxicillin–clavulanic acid, ampicillin, and nalidixic acid was observed among the gram-negative bacteria. Resistant to penicillin G, erythromycin, amoxicillin–clavulanic acid, and ampicillin, cefoxitin was shown in gram-positive bacteria. Moreover, a significant multi-drug resistance was observed in the isolated bacteria. These calls for strengthening the existing infection prevention and antibiotic stewardship program with the application of strict follow up to minimize bacterial contamination of medical equipment and inanimate surfaces.

## Limitation

This study limited to carry out the extended beta-lactam spectrum.

## Data Availability

Not applicable.

## Abbreviations

CoNS: coagulase-negative staphylococcus
CLSI: Clinical and Laboratory Standards Institute
ICU: intensive care unit
IP: infection prevention
MDR: multidrug resistance
SPSS: Statistical Package for Social Sciences
